# A mobile phone application for the prevention of type 2 diabetes in Malaysian women with gestational diabetes mellitus (MYGODDESS): A feasibility randomised controlled trial

**DOI:** 10.1111/dme.70257

**Published:** 2026-03-10

**Authors:** Madeleine Benton, Kimberley Goldsmith, Siew Mooi Ching, Angus Forbes, Iliatha Papachristou Nadal, Helen R. Murphy, Nur Hafizah Mahamad Sobri, Barakatun‐Nisak Mohd Yusof, Nurul Iftida Basri, Irmi Zarina Ismail, Khalida Ismail, Boon How Chew

**Affiliations:** ^1^ Department of Psychological Medicine, Institute of Psychiatry Psychology and Neuroscience King's College London London UK; ^2^ Department of Biostatistics & Health Informatics King's College London London UK; ^3^ Department of Family Medicine, Faculty of Medicine and Health Sciences Universiti Putra Malaysia Selangor Malaysia; ^4^ Division of Care in Long‐Term Conditions King's College London London UK; ^5^ Department of Medicine University of East Anglia Norfolk UK; ^6^ Department of Nutrition and Dietetics Universiti Putra Malaysia Selangor Malaysia; ^7^ Department of Obstetrics and Gynaecology Universiti Putra Malaysia Selangor Malaysia; ^8^ Clinical Research Unit Hospital Pengajar Universiti Putra Malaysia Serdang Malaysia

**Keywords:** diabetes, feasibility study, mobile application, pregnancy

## Abstract

**Aims:**

The prevalence of gestational diabetes mellitus (GDM) in Malaysia is estimated at 9–18%. Although GDM is associated with increased and potentially modifiable risk of developing type 2 diabetes, the effectiveness of diabetes prevention interventions (DPI) post‐GDM in this setting is unclear. To evaluate the feasibility of conducting a future full‐scale, two‐arm, parallel, randomised controlled trial (RCT) of a DPI in women with GDM set in Malaysia.

**Methods:**

Women in both arms received usual GDM care. Women in the intervention arm also received modules on diet, physical activity, and mental health via a mobile application, over six months post‐partum, plus dietitian‐led group sessions and motivational text messages. The primary feasibility outcomes included the proportion of women who consented, were eligible and randomised and provided outcome data. We measured biomedical and mental health outcomes for a full‐scale RCT at four time points: baseline before randomisation (approximately 30 weeks' gestation), 36 weeks' gestation and 3‐ and 6‐months postpartum.

**Results:**

We screened 660 women with GDM, 294 (45%) consented for eligibility screening, of whom 164 (24.9%) were eligible and 60 (9%) consented and were randomised. The proportion who completed biomedical outcomes was 85% at each follow‐up. There was no treatment effect on any other biomedical outcomes or secondary outcomes.

**Conclusions:**

The participation rate was in keeping with previous DPI trials and the attrition rate was low, suggesting it is feasible to conduct a full‐scale RCT.

**What's new?**

**What is already known?**

- Women with prior gestational diabetes mellitus are at high risk of developing type 2 diabetes, but evidence on delivering digital prevention interventions in low‐ and middle‐income countries is limited

**What this study has found?**

- A digital diabetes prevention intervention for Malaysian women with GDM was feasible and acceptable, with good engagement, recruitment, and follow‐up rates.

**What are the implications of the study?**

- The study supports a future full‐scale RCT and provides practical insights into trial design, including the timing of recruitment and postpartum follow‐up logistics in Malaysia.

## 1 INTRODUCTION

The pooled global prevalence of gestational diabetes mellitus (GDM) is estimated at 14.0%, with a regional prevalence of around 7% in North America and Europe and 21% in South‐East Asia.^1^ The prevalence of GDM in Malaysia is estimated between 9.3% and 18.5%.^2^, ^3^ GDM is associated with a nine‐fold increased risk of type 2 diabetes (T2D) for the mother^4^ and increased risk of obesity and T2D for the infant.^5^, ^6^ The post‐partum period therefore represents a key window for modifying these risks, as it is characterised by increased contact with health services and provides a convenient opportunity for both mother and infant to receive additional support.

Intensive diabetes prevention interventions (DPI) are structured programmes designed to reduce the risk of progression to T2D typically through change to diet, physical activity, and weight. They are often delivered by health care professionals and can reduce the risk of T2D by around 50% in the general population,^7^, ^8^, ^9^ but there is less evidence for their effectiveness in women with GDM.^10^, ^11^ The most recent meta‐analysis of 24 RCTs of DPIs for women with GDM reported a pooled 19% reduction in the incidence of T2D.^12^

Digital DPIs present an alternative method for delivering high intensity lifestyle interventions via mobile applications, web‐based platforms, or remote coaching. Their advantage is that they can increase reach and reduce costs. They have been evaluated and implemented in several high‐income settings but not in low‐ and middle‐income settings. Digital DPIs have been associated with pooled estimates of approximately 5% weight loss.^13^ In many low‐ and middle‐income settings, such as Malaysia, there is widespread use of smartphones even in remote regions and across the socioeconomic spectrum. Digital DPIs may offer additional advantages beyond low cost, including improved accessibility for post‐partum women with competing childcare and work demands and large geographical distances between health services, scalability within resource‐constrained health systems, and potential integration with existing maternal and primary care services. Scalability is important in Asian populations, where the prevalence of GDM is increasing and the absolute burden of risk for T2D is high. There have been a considerable number of RCTs of DPIs for women with GDM; however, there is limited evidence for the effectiveness of digital DPIs in this population on T2D risk reduction.^13^ For instance, in a systematic review of the intervention components of 24 RCTs of DPI post GDM, the format of delivery was in‐person (*n* = 8), digital (*n* = 7) or hybrid (*n* = 9).^14^ The intervention duration varied from 12 weeks to 6 years.^14^ Most studies (*n* = 22, 92%) were conducted in high‐income countries with only one study set in Malaysia. This RCT compared conventional dietary advice to additional low glycaemic index (GI) dietary education in 77 women with previous GDM and observed women receiving the low‐GI intervention had greater reductions in body weight, BMI and waist‐to‐hip ratio over 6 months compared with conventional advice alone.^15^ However, this intervention was delivered in person and therefore more costly to scale than a digital DPI and focused on dietary modification rather than multi‐component lifestyle change.

The aim of this study, titled ‘MalaYsian GestatiOnal Diabetes and prevention of DiabetES Study’ (MYGODDESS), is to evaluate the feasibility of conducting a future full scale RCT of a digital DPI post‐GDM.

## 2 METHODS

### 2.1 Design

**FIGURE 1:**
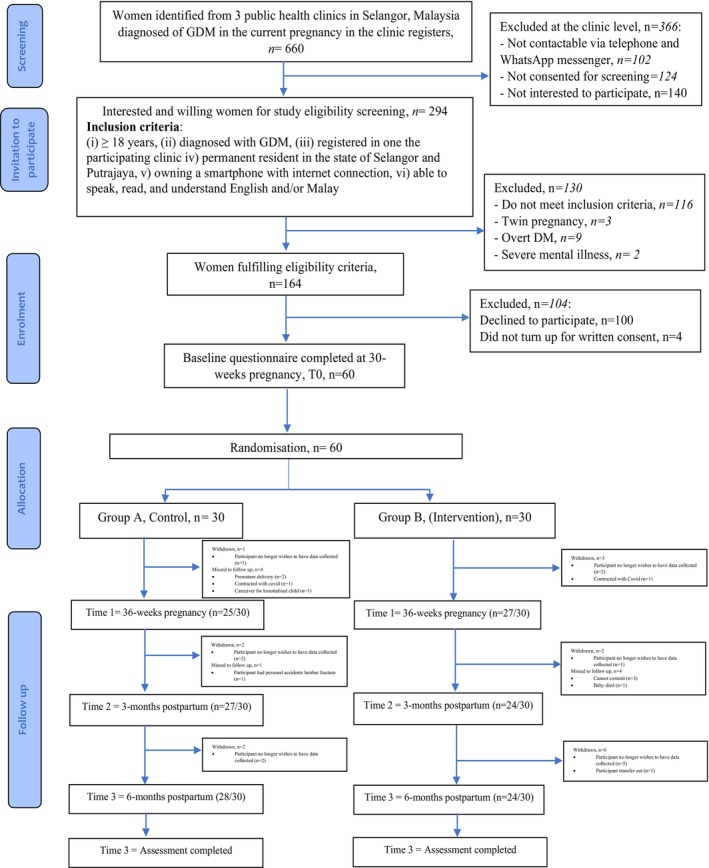
CONSORT flow diagram.

MYGODDESS is a feasibility two‐arm, parallel, individual RCT (clinicaltrials.gov NCT05204706). A detailed description of the trial protocol is published elsewhere.^16^ Ethical approval was granted by the Malaysian Ministry of Health's Medical Research and Ethics Committee (NMRR‐21‐1667‐60,212). Reporting followed the Consolidated Standards of Reporting Trials (CONSORT) 2010 Statement: extension to randomised pilot and feasibility trials (www.consort‐spirit.org/) (Figure 1).

### 2.2 Setting

The study was set in three primary care clinics in Selangor state and the federal territory of Putrajaya which together have a population of 6.77 million residents, of which 57.1%, 28.6%, 13.3% and 0.8% were of Malay, Chinese, Indian and other ethnicity respectively. Malaysia is a middle‐income nation in the Organisation for Economic Co‐operation and Development list.

In the Malaysian public health system, GDM management follows a decentralised model anchored in public health clinics (Klinik Kesihatan), which serve as the primary entry point for antenatal care. Upon diagnosis via an Oral Glucose Tolerance Test (OGTT), patients are managed at the Maternal and Child Health unit in these clinics under the supervision of Family Medicine Specialists. The clinical pathway typically initiates with Medical Nutrition Therapy, often delivered through group counselling, and if required, pharmacotherapy, predominantly Metformin or Insulin. A distinctive feature of this setting is the monitoring protocol: rather than strictly relying on daily home self‐monitoring, many patients attend scheduled clinics for ‘Blood Sugar Profiles’ (testing performed by nurses throughout a single day) to assess management. Patients requiring Insulin or those with obstetric complications are then referred upwards to tertiary hospitals for specialised management by Obstetricians and Endocrinologists.

### 2.3 Study population and sample selection

The study population was women with a current diagnosis of GDM defined as fasting blood glucose ≥5.1 mmol/1 or 2‐h postprandial ≥7.8 mmol/1 following the 75 g OGTT at booking or early as possible for women at risk of developing GDM and for women age ≥25 years with no other risk factors at 24–28 weeks of gestation, based on national guidance ‘Management of Diabetes in Pregnancy.^17^ The inclusion criteria were: age ≥ 18 years; permanent residents in the state of Selangor or Putrajaya; registered in one of the study primary care clinics; owned a smartphone (iOS 11 or above or Android) with internet connection; able to communicate in English and/or Malay. The exclusion criteria were: twin pregnancy; diagnosed or treated as type 1 or 2 diabetes or other type of diabetes; a physical disability that would prevent any increased uptake of physical exercise; severe mental illness (psychosis, bipolar, substance dependence or active suicidal ideation); currently participating in a weight loss program or DPI.

### 2.4 Enrolment procedure

The clinicians at the primary care clinics introduced the study to potentially eligible women who were diagnosed with GDM during their current pregnancy. Due to the Covid‐19 restrictions, participants who expressed an interest in participating received further information about the study and pre‐screening via a phone call. Those who were eligible were invited to give informed consent in person. Those who consented were randomised after completing all the baseline assessments (time 0) at 30 weeks of gestation with a window period of one week.

### 2.5 Randomisation procedure

A randomisation list was generated by an online generator (https://www.sealedenvelope.com, Sealed Envelope Ltd. 2021) using random permuted blocks of two and four with allocations to usual care or DPI in a 1:1 ratio. The randomisation list was generated, kept, and password protected by the Clinical Research Unit (CRU), Universti Putra Malaysia, whose research officers were the only individuals with access to the list. Following baseline assessment which included prospective three days of dietary records, a research member contacted the CRU to obtain the randomised allocation for each participant.

### 2.6 Control

Patients in the control arm received usual care as described in Management of Diabetes in Pregnancy.^17^ This included self‐monitoring blood glucose and lifestyle advice (diet, physical activity, optimal body weight) by a multidisciplinary team in primary care consisting of a family physician, two non‐specialist medical doctors, nurse specialists in obstetrics, midwives, pharmacists, dietitians or diabetes educator.

### 2.7 Intervention

In addition to usual care, the intervention consisted of three components: access to the mobile app called MyManis (translated from Malay to English as My Sweet Thing); group sessions delivered by a dietician using motivational interviewing skills; and automated motivational text messages. The intervention was underpinned by the Information‐Motivation‐Behavioural Skills model of behaviour change and motivational interviewing (MI).^18^

The app consisted of the following self‐directed modules: health education about GDM and advice and support on changes in diet, physical activity and mental health from pregnancy to post‐partum. We designed culture‐specific affordable recipes; antenatal and postnatal exercise videos delivered by pregnant women; and information about GDM, mental health and eliciting social support based on feedback from patient participation and involvement. Once the app had been installed, participants created an account with their name, date of birth, current gestational week, and height and weight. The app was available for the duration of the study.

Women were also invited to join eight group support sessions lasting one‐hour, delivered fortnightly via a virtual Zoom meeting facilitated by a dietician trained in MI. Each session focused on a specific topic related to barriers to changing diet and physical activity, breastfeeding, and understanding the nature of GDM. The dietician had attended an online 10 h course over 6 weeks, ‘Foundations of MI’ (https://psychwire.com/motivational‐interviewing/mi‐foundational) delivered by qualified MI trainers, followed by 18 supervision sessions from two MI therapists based in the UK and in Malaysia. It was not possible to test the dietician's competency because there were no Malay speaking therapists trained in rating MI skills at the time of the study.

Between the sessions, the women received one automated motivational text message three times a week via mobile phone. The messages were selected, culturally adapted and translated from DPI app that had been developed in the UK for people with prediabetes.^19^ Each message was unique and categorised as dietary, physical activity, mental health or educational.

### 2.8 Development of the intervention (MyManis app)

The development of the MyManis app followed a rigorous, evidence‐based approach guided by the Design and Development, Testing Early Iterations, Testing for Effectiveness, and Integration and Implementation (DOTTI) framework. The development was conducted in three phases. Firstly, a needs assessment was conducted, including semi‐structured interviews with *n* = 12 Malaysian dietitians to understand current practices, challenges, and the need for digital tools, alongside workshops with women who had previous GDM. Co‐design workshops were then conducted where the app content was developed with healthcare professionals (dietitians, obstetricians, endocrinologists, family medicine specialists, etc.) and women with GDM, alongside the mobile app developers. The app underwent Alpha testing (internal team) and Beta testing (*n* = 13 women with GDM and *n* = 12 HCPs) to refine usability, fix technical bugs, and ensure cultural relevance. The final phase involved a larger‐scale evaluation with *n* = 77 practicing dietitians nationwide to assess usability, using the mHealth App Usability Questionnaire^20^ and quality, using the Mobile App Rating Scale^21^ in a real‐world clinical setting.

### 2.9 Blinding

The participants could not be blinded to allocation as the nature of the intervention involved interaction with the app and talking therapy but the research team collecting and analysing the data were protocolised to be blinded.

### 2.10 Feasibility outcome

The primary feasibility outcomes were to estimate:

1. the proportion of women who (a) consented to screening for eligibility, (b) were eligible and (c) randomised from those identified from the clinical registers (study population).
2. the proportion of women who received the DPI in the intervention arm, to be defined by a self‐report questionnaire which we designed for this study.
3. the proportion of women who (a) withdrew or (b) were lost to follow‐up as defined by missing clinical anthropometric outcomes at each time point;
4. the rate of recruitment defined as number of women randomised per month per study site.

The clinical outcomes were collected at four time points: time 0 representing the baseline (30 weeks gestation), time 1 (36 weeks gestation), time 2 (3 months post‐partum) and time 3 (6 months post‐partum). In addition, sociodemographic factors were collected at baseline as follows: age, self‐report ethnicity (Malay, Chinese, Indian or other), education attainment, average household income per month, smoking status was measured using a single item measure with response options including current smoker, previous smoker, and never smoked. Alcohol intake was measured using the first question of the Alcohol Use Disorders Identification Test.

Anthropometric outcomes were collected at all four time points as follows: weight (kg), height (time 0 only), body mass index (BMI) (kg/m^2^), and body fat percentage.

Biomedical outcomes were collected at all four time points: systolic and diastolic blood pressure (mmHg); total cholesterol (mmol/L); LDL‐cholesterol (mmol/L); HDL‐cholesterol (mmol/L); HbA1c (mmol/mol); fasting plasma glucose (mmol/L); insulin resistance (HOMA‐IR); 75 g OGTT 2‐h postprandial (mmol/L) (time 3 only).

The psychological outcomes were measured at time 0 and time 3 as follows: Self‐efficacy for Exercise Scale (SES)^22^; the Maternal Antenatal Attachment Scale (MAAS) measured at time 0 and the Maternal Postnatal Attachment Scale (MPAS) measured at time 3^23^; the Patient Health Questionnaire‐9 (PHQ‐9).^24^

Lifestyle outcomes were measured at time 0 and time 3 as follows: dietary intake was assessed using standardised multiple‐pass 24‐h dietary recall for 3 days (2 weekdays and 1 weekend day)^25^; Short‐Form International Physical Activity Questionnaire (SF‐IPAQ)^26^; average daily step count over seven days was derived from participant's pedometer function in participant's smartphone; infant feeding practice was assessed by one item with five options ranging from fully breastfeeding, almost breastfeeding (about four breastfeeds of five feeds/day), mixed feeding, mostly bottle feeding (about one breastfeed of 5 feeds/day) to fully bottle feeding at time 3 only.

Clinical obstetric variables were collected from the medical records and described in the protocol paper and reported in Appendix 1.

The uptake of the DPI was assessed by a self‐report questionnaire which included dates and times of day for each log in, total time spent on the app and time spent on each module of the app.

### 2.11 Data collection

Data collection was a coordinated effort led by the PI and research team, who managed the overall logistics, distributed digital surveys for psychosocial and lifestyle assessments, and conducted on‐site data collection, including the qualitative interviews. Site‐specific researchers conducted the baseline assessments and group allocations, while clinical staff with the staff nurses at the health clinics were responsible for venepuncture. Where necessary, the clinics' Medical and Health Officers (primary care physicians with undergraduate medical qualification) assisted in validating the biomedical outcome data.

For dietary assessment, a blinded methodology was employed to minimise bias. Participants submitted meal photos over three days via WhatsApp to the Clinical Research Unit (CRU) who acted as independent data managers. CRU officers de‐identified these submissions by replacing study IDs with ‘Diet IDs’ before forwarding them to the lead researcher for nutrient analysis. Once the analysis was complete, the CRU officers re‐matched the results to the master study list, ensuring the researcher remained blinded to the participants' identities during the evaluation.

Antenatal Phase (Time 0 and Time 1): The baseline data collection at Time 0 (24–30 weeks gestation) was selected to align directly with the standard Malaysian diagnosis window for GDM, ensuring that recruitment occurred immediately following a confirmed diagnosis. This timing provided a critical 8–10 week window for participants to engage with the app's educational modules before delivery. The subsequent follow‐up at Time 1 (36 weeks gestation) served as a vital late‐pregnancy checkpoint to evaluate the immediate efficacy of the intervention on dietary behaviour and mental health prior to birth and to support engagement post‐partum. Crucially, this time point also functioned as a safety monitoring mechanism to assess maternal weight gain and screen for complications like pre‐eclampsia before birth.

Postnatal Phase (Time 2 and Time 3): While the standard Ministry of Health protocol requires an OGTT at 6 weeks, positioning data collection at 3 months allowed researchers to retrospectively verify attendance at this mandatory test, a primary feasibility outcome, while avoiding the logistical issues of the immediate newborn period. Furthermore, 3 months typically marks the end of the traditional confinement period in Malaysia (pantang), offering a more realistic baseline to assess outcomes once regular daily routines resume. Finally, Time 3 at 6 months postpartum was chosen to evaluate the medium‐term sustainability of the intervention. This point specifically addresses the local ‘postnatal gap’, determining whether the app could motivate women to maintain diabetes prevention behaviours (diet and physical activity) well beyond the period of active medical supervision.

### 2.12 Sample size

The aim was to recruit *n* = 60 women as sufficient to estimate the standard deviation, from which the sample size for an appropriately powered full‐scale RCT could be estimated.^27^

### 2.13 Statistical analyses

Analyses were performed using SPSS version 26 (SPSS version 26; SPSS IBM, New York, USA) following the intention‐to‐treat principle. For each feasibility outcome, we calculated proportions (percentage) with confidence intervals (CIs).

The clinical outcomes were summarised using summary statistics, overall and by randomised arm at each outcome time point. Normally distributed continuous variables were summarised using the mean and standard deviation (SD), skewed continuous variables using the median and interquartile range, while categorical variables were reported using frequencies and proportions.

As the majority of the outcomes were continuous and repeatedly measured, we estimated the mean difference between the arms using linear mixed‐effects models with a random intercept at the participant level to account for repeated measures. The repeatedly measured outcomes were the dependent variables, with the independent variables being trial arm, time point, and a trial arm by time interaction term to extract differences at different time points. As this was a feasibility study, we did not present p‐values for differences between the arms.^28^ Effect sizes were calculated by dividing the mean differences by respective baseline SDs using the whole sample, to enable comparisons across measures and time points in baseline SD units.

### 2.14 Protocol violations

As a result of delays and restrictions to recruitment secondary to Covid‐19 lockdown, the follow‐up period was reduced from 12 months to six months. We were unable to analyse the self‐report questionnaire on the uptake of the DPI as there was a technical problem in retrieving it from the UPM server. However, we were able to collect data on the group sessions involved in the DPI which had a mean attendance of three women per session (range = 0–6 women per session).

## 3 RESULTS

### 3.1 Feasibility outcomes

Study recruitment took place between 10 February 2022 and 2 November 2023. Figure 1 describes the study flow and the feasibility outcomes. Of the 660 women identified by the clinic (the study population) as potentially eligible, 366 were excluded, the majority because they declined the invitation to participate. Of the remaining 294 (45% of study population) who gave consent for screening by telephone, 130 were not eligible. Of the 164 (24.9% of the study population) who met the eligibility criteria, 60 (9% of study population) participants consented and completed the baseline assessment at time 0 and randomised. The proportion of those randomised who completed clinical outcomes at times 1, 2 and time 3 was 86%, 85% and 86.0% respectively.

Table 1 summarises the baseline (T0) characteristics which is at the time of diagnosis of GDM. Mean (SD) age of the sample was 31.3 (4.2) years. The majority were of Malay ethnicity, had a tertiary education and had an average household income range RM3000–RM4999 per month (equivalent to US dollars 670‐1118) representing a lower income group.^29^ None of the participants were current smokers or reported any alcohol intake. The proportion of women randomised did vary by study site.

**TABLE 1:** Baseline sociodemographic characteristics of MYGODDESS trial participants at 30 weeks' gestation.

Baseline characteristic	Control group (*N* = 30)	Intervention group (*N* = 30)	Overall *N* = 60
Age, years (mean ± SD)	32.2 ± 3.9	30.5 ± 4.3	31.3 ± 4.2
Ethnicity, *n* (%)			
Malay	26 (86.7)	23 (76.7)	49 (82.0)
Chinese	2 (6.7)	6 (20.0)	8 (13.0)
Indian	1 (3.3)	1 (3.3)	2 (3.0)
Indigenous and other	1 (3.3)	0 (0.0)	1 (2.0)
Highest education level, *n* (%)			
No formal education	0 (0.0)	0 (0.0)	0 (0.0)
Primary	0 (0.0)	1 (2.0)	1 (2.0)
Secondary	3 (10.0)	7 (23.3)	10 (16.0)
Tertiary	27 (73.3)	22 (73.3)	49 (82.0)
Employment status, *n* (%)			
Employed	22 (73.3)	21 (70.0)	43 (72.0)
Unemployed	7 (23.3)	6 (20.0)	13 (21.0)
Retired	0 (0.0)	0 (0.0)	0 (0.0)
Other (casual worker, student, military)	1 (3.3)	3 (10.0)	4 (7.0)
Household income per month in Malaysian ringgit, *n* (%)			
<RM 1000	0 (0.0)	1 (3.3)	1 (2.0)
RM 1000–2999	9 (30.0)	6 (20.0)	15 (25.0)
RM 3000–4999	11 (36.7)	13 (43.3)	24 (39.0)
RM 5000–10,000	8 (26.7)	8 (26.7)	16 (27.0)
>RM 10,000	2 (6.7)	2 (6.7)	4 (7.0)
Smoking status, *n* (%)			
Non‐smoker	30 (50.0)	30 (50.0)	60 (100.0)
Active, ex, passive smoker	0 (0.0)	0 (0.0)	0 (0.0)
Alcohol intake, *n* (%)			
Never	30 (50.0)	30 (50.0)	60 (100.0)
One or few times per month or more	0 (0.0)	0 (0.0)	0 (0.0)
Place of antenatal care			
Klinik Kesihatan Seri Kembangan	7 (23.3)	13 (43.3)	20 (33.4)
Klinik Kesihatan Puchong Batu 14	13 (43.3)	11 (36.7)	24 (40.0)
Klinik Kesihatan Putrajaya Presint 9	8 (26.7)	3 (10.0)	11 (18.3)
Missing	2 (6.7)	3 (10.0)	5 (8.3)

Table 2 summaries the average values for potential outcomes at baseline and at each of the three follow ups. Importantly, the cohort was on average borderline for obesity and had excess body fat percentage. The average PHQ‐9 score represented mild or subthreshold depression. The follow‐up rates for the biomedical outcomes were slightly higher than for self‐report questionnaires at 85% and around 70% respectively. For infant feeding practices at six months post‐partum, just over a third were exclusively breastfeeding.

**TABLE 2:** Anthropometry, biochemical, psychological and lifestyle profiles of the participants of the MYGODDESS trial at Time 0 (baseline, 30 weeks gestation), Time 1 (36 weeks gestation), Time 2 (3‐month postpartum) and Time 3 (6‐month post‐partum).

Variables	Statistical quantity	Control group (*N* = 30)	Intervention group (*N* = 30)	Overall (*N* = 60)
Body mass index (kg/m^2^)				
T0 baseline	Mean (SD)	29.3 (6.4)	29.7 (5.2)	29.5 (5.8)
	# Complete (%)	30/30 (100.0)	30/30 (100.0)	60/60 (100.0)
T1 36 weeks gestation	Mean (SD)	30.7 (6.7)	30.6 (4.9)	30.6 (5.7)
	# Complete (%)	25/30 (83.3)	27/30 (90.0)	52/60 (86.7)
T2 3‐month postpartum	Mean (SD)	27.4 (6.4)	27.6 (5.5)	27.5 (5.9)
	# Complete (%)	27/30 (90.0)	23/30 (76.7)	50/60 (83.3)
T3 6‐month post‐partum	Mean (SD)	27.6 (6.5)	28.1 (5.5)	27.8 (6.0)
	# Complete (%)	28/30 (93.3)	23/30 (76.7)	51/60 (85.0)
Body fat percentage (%)				
T0 baseline	Mean (SD)	42.1 (6.9)	44.9 (6.0)	43.5 (6.6)
	# Complete (%)	30/30 (100.0)	30/30 (100.0)	60/60 (100.0)
T1 36 weeks gestation	Mean (SD)	43.3 (7.8)	44.1 (5.4)	43.7 (6.6)
	# Complete (%)	25/30 (83.3)	27/30 (90.0)	52/60 (86.7)
T2 3‐month postpartum	Mean (SD)	37.3 (8.4)	38.9 (7.8)	38.0 (8.1)
	# Complete (%)	27/30 (90.0)	23/30 (76.7)	50/60 (83.3)
T3 6‐month post‐partum	Mean (SD)	37.9 (8.1)	40.2 (7.2)	38.9 (7.7)
	# Complete (%)	28/30 (93.3)	23/30 (76.7)	51/60 (85.0)
HbA1c (%)				
T0 baseline	Mean (SD)	5.3 (0.3)	5.3 (0.3)	5.3 (0.3)
	# Complete (%)	30/30 (100.0)	30/30 (100.0)	60/60 (100.0)
T1 3‐months post‐partum	Mean (SD)	5.4 (0.4)	5.5 (0.3)	5.4 (0.4)
	# Complete (%)	25/30 (83.3)	27/30 (90.0)	50/60 (83.3)
T3 6‐month post‐partum	Mean (SD)	5.5 (0.3)	5.5 (0.4)	5.5 (0.4)
	# Complete (%)	28/30 (93.3)	23/30 (76.7)	51/60 (85.0)
Fasting blood glucose (mmol/L)				
T0 baseline	Mean (SD)	4.3 (0.4)	4.4 (0.4)	4.3 (0.4)
	# Complete (%)	30/30 (100.0)	30/30 (100.0)	60/60 (100.0)
T1 3‐months post‐partum	Mean (SD)	4.2 (0.6)	4.4 (0.4)	4.3 (0.5)
	# Complete (%)	25/30 (83.3)	27/30 (90.0)	52/60 (86.7)
T3 6‐month post‐partum	Mean (SD)	4.6 (0.4)	4.6 (0.5)	4.6 (0.4)
	# Complete (%)	28/30 (93.3)	23/30 (76.7)	51/60 (85.0)
Fasting serum insulin (mIU/L)				
T0 baseline	Mean (SD)	9.8 (5.3)	9.7 (4.8)	9.8 (5.0)
	# Complete (%)	30/30 (100.0)	30/30 (100.0)	60/60 (100.0)
T1 3‐months post‐partum	Mean (SD)	11.7 (8.3)	10.0 (5.1)	10.8 (6.8)
	# Complete (%)	25/30 (83.3)	27/30 (90.0)	52/60 (86.7)
T3 6‐month post‐partum	Mean (SD)	8.3 (7.4)	7.2 (4.4)	7.8 (6.2)
	# Complete (%)	28/30 (93.3)	23/30 (76.7)	51/60 (85.0)
Homeostatic Model Assessment for Insulin Resistance				
T0 baseline	Mean (SD)	1.9 (1.1)	1.9 (1.0)	1.9 (1.0)
	# Complete (%)	30/30 (100.0)	30/30 (100.0)	60/60 (100.0)
T1 3‐months post‐partum	Mean (SD)	2.3 (2.0)	2.0 (1.1)	2.1 (1.6)
	# Complete (%)	25/30 (83.3)	27/30 (90.0)	52/60 (86.7)
T3 6‐month post‐partum	Mean (SD)	1.9 (1.7)	1.7 (1.2)	1.8 (1.5)
	# Complete (%)	28/30 (93.3)	23/30 (76.7)	51/60 (85.0)
2‐h oral glucose tolerance test (OGTT)^a^				
T3 6‐month post‐partum	Mean (SD)	5.6 (1.2)	5.6 (1.6)	5.6 (1.3)
	# Complete (%)	28/30 (93.3)	23/30 (76.7)	51/60 (85.0)
Systolic blood pressure (Hgmm)				
T0 baseline	Mean (SD)	109.0 (9.9)	111.6 (12.4)	110.3 (11.2)
	# Complete (%)	30/30 (100.0)	30/30 (100.0)	60/60 (100.0)
T1 3‐months post‐partum	Mean (SD)	111.8 (10.0)	110.8 (10.4)	111.3 (10.1)
	# Complete (%)	25/30 (83.3)	27/30 (90.0)	52/60 (86.7)
T3 6‐month post‐partum	Mean (SD)	117.5 (10.8)	113.4 (8.7)	115.7 (10.0)
	# Complete (%)	28/30 (93.3)	23/30 (76.7)	51/60 (85.0)
Diastolic blood pressure (Hgmm)				
T0 baseline	Mean (SD)	66.3 (7.9)	70.4 (10.0)	68.4 (9.1)
	# Complete (%)	30/30 (100.0)	30/30 (100.0)	60/60 (100.0)
T1 3‐months post‐partum	Mean (SD)	70.1 (7.6)	68.8 (8.0)	69.4 (7.7)
	# Complete (%)	25/30 (83.3)	27/30 (90.0)	52/60 (86.7)
T3 6‐month post‐partum	Mean (SD)	78.9 (8.7)	74.2 (9.5)	76.8 (9.3)
	# Complete (%)	28/30 (93.3)	23/30 (76.7)	51/60 (85.0)
Total cholesterol (mmol/L)				
T0 baseline	Mean (SD)	6.8 (1.1)	6.6 (1.2)	6.7 (1.1)
	# Complete (%)	30/30 (100.0)	30/30 (100.0)	60/60 (100.0)
T1 3‐months post‐partum	Mean (SD)	7.2 (1.3)	6.7 (1.0)	7.0 (1.2)
	# Complete (%)	25/30 (83.3)	27/30 (90.0)	52/60 (86.7)
T3 6‐month post‐partum	Mean (SD)	5.2 (0.9)	4.9 (0.8)	5.0 (0.9)
	# Complete (%)	28/30 (93.3)	23/30 (76.7)	51/60 (85.0)
High density lipoprotein cholesterol (mmol/L)				
T0 baseline	Mean (SD)	1.0 (0.0)	1.0 (0.0)	1.0 (0.0)
	# Complete (%)	30/30 (100.0)	30/30 (100.0)	60/60 (100.0)
T1 3‐months post‐partum	Mean (SD)	2.0 (0.5)	2.0 (0.5)	2.0 (0.5)
	# Complete (%)	25/30 (83.3)	27/30 (90.0)	52/60 (86.7)
T3 6‐month post‐partum	Mean (SD)	1.6 (0.4)	1.7 (0.7)	1.7 (0.6)
	# Complete (%)	28/30 (93.3)	23/30 (76.7)	51/60 (85.0)
Low Density Lipoprotein Cholesterol, LDL‐C (mmol/L)				
T0 baseline	Mean (SD)	3.7 (1.3)	3.5 (1.0)	3.6 (1.2)
	# Complete (%)	30/30 (100.0)	30/30 (100.0)	60/60 (100.0)
T1 3‐months post‐partum	Mean (SD)	4.0 (1.3)	3.4 (1.1)	3.7 (1.2)
	# Complete (%)	24/30 (80.0)	27/30 (90.0)	51/60 (85.0)
T3 6‐month post‐partum	Mean (SD)	3.1 (0.7)	2.7 (0.7)	2.9 (0.7)
	# Complete (%)	28/30 (93.3)	23/30 (76.7)	51/60 (85.0)
Self‐Efficacy for Exercise				
T0 baseline	Mean (SD)	45 (11)	45 (11)	45 (11)
	# Complete (%)	30/30 (100.0)	28/30 (93.3)	58/60 (96.7)
T3 6‐month post‐partum	Mean (SD)	43 (16)	50 (18)	46 (17)
	# Complete (%)	24/30 (80.0)	19/30 (63.3)	43/60 (71.7)
Maternal Antenatal/Postnatal Attachment Scale, MAAS/MPAS				
T0 baseline (MAAS)	Mean (SD)	29.6 (2.6)	32.9 (12.1)	31.2 (8.8)
	# Complete (%)	30/30 (100.0)	28/30 (93.3)	56/60 (100.0)
T3 6‐month post‐partum (MPAS)	Mean (SD)	3.7 (0.3)	3.7 (0.3)	3.7 (0.3)
	# Complete (%)	24/30 (80.0)	19/30 (63.3)	43/60 (71.7)
Patient Health Questionnaire‐9				
T0 baseline	Mean (SD)	7 (4)	8 (4)	7 (4)
	# Complete (%)	30/30 (100.0)	28/30 (93.3)	58/60 (96.7)
T3 6‐month post‐partum	Mean (SD)	6 (4)	7 (4)	6 (4)
	# Complete (%)	24/30 (80.0)	19/30 (63.3)	43/60 (71.7)
24‐h dietary recall (Total Energy Intake), g/day				
T0 baseline	Median (IQR)	1384 (464)	1331 (499)	1349 (497)
	# Complete (%)	25/30 (83.3)	25/30 (83.3)	50/60 (83.3)
T3 6‐month post‐partum	Median (IQR)	1231 (490)	884 (303)	1160 (466)
	# Complete (%)	19/30 (63.3)	13/30 (43.3)	32/60 (53.3)
International Physical Activity Questionnaire‐Short Form (IPAQ‐SF), total mets per mins/week				
T0 baseline	Median (IQR)	1478 (3178)	899 (1999)	1404 (1902)
	# Complete (%)	30/30 (100.0)	30/30 (100.0)	60/60 (100.0)
T3 6‐month post‐partum	Median (IQR)	1544 (1826)	784 (1149)	1272 (1556)
	# Complete (%)	24/30 (80.0)	20/30 (66.7)	44/60 (73.3)
Median daily step count				
T0 baseline	Median (IQR)	1888 (1741)	2116 (3643)	2000 (2823)
	# Complete (%)	30/30 (100.0)	30/30 (100.0)	60/60 (100.0)
T3 6‐month post‐partum	Median (IQR)	1058 (2406)	2000 (4018)	1500 (2400)
	# Complete (%)	24/30 (80.0)	19/30 (63.3)	43/60 (71.7)
Infant feeding practice T3 6‐month post‐partum				
Fully breast feeding	*N* (%)	12 (40.0)	11 (36.7)	23 (38.4)
Mostly breast feeding	*N* (%)	0 (0)	0 (0)	0 (0)
Mixed feeding	*N* (%)	5 (16.7)	9 (30.0)	14 (23.3)
Mostly formula feeding	*N* (%)	0 (0)	0 (0)	0 (0)
Fully formula feeding	*N* (%)	2 (6.7)	4 (13.3)	6 (10.0)
Missing	*N* (%)	11 (36.7)	6 (20.0)	17 (28.3)
	# Complete (%)	30/30 (100.0)	30/30 (100.0)	60/60 (100%)

*Note*: T0 (Time 0 (baseline, 30 weeks gestation)); T1 (Time 1 (36 weeks gestation)). T2 (Time 2 (3‐month postpartum)); T3 (Time 3 (6‐month post‐partum)).

^a^ OGTT was conducted at only one time point.

Table 3 reports the mean differences between the control and intervention groups comparing the value of each clinical outcome at T0 (baseline) with its value at each of the three follow up time points. Although not powered to estimate statistically significant effects, there were no differences in any of the outcomes, although there was a trend for the systolic and diastolic blood pressure to have reduced by approximately 5 mmHg. There was a small increase in self‐efficacy for exercise and in reduction in total energy intake at six months post‐partum. There were no findings suggesting that the MYGODDESS intervention worsened health.

**TABLE 3:** Estimated mean difference between control and intervention groups comparing clinical outcomes between Time 0 (baseline) with Time 1 (36 weeks gestation), Time 2 (3‐month postpartum) and Time 3 (6‐month post‐partum).

Variables	36‐weeks pregnancy (Time 1)		3‐months postpartum (Time 2)		6‐months postpartum (Time 3)	
	Mean difference	95% CI	Mean difference	95% CI	Mean difference	95% CI
Anthropometry						
Body mass index (kg/m^2^) (*n* = 51)	0.32	[−0.60, 1.23]	0.10	[−0.83, 1.03)	0.07	[−0.85, 1.00]
Body fat percentage (%) (*n* = 51)	−0.40	[−3.53, 2.72]	0.05	[−3.12, 3.22]	0.55	[−2.61, 3.71]
Biomedical						
Haemoglobin A1C (HbA1c) (%) (*n* = 34)	0.02	[−0.11, 0.15]	NA	NA	−0.08	[−0.22, 0.05]
Fasting blood glucose (mmol/L) (*n* = 34)	0.09	[−0.15, 0.33]	NA	NA	0.07	[−0.17, 0.32]
Fasting serum insulin (*n* = 34)	−1.89	[−5.30, 1.52]	NA	NA	−0.88	[−4.34, 2.57]
Insulin resistance, HOMA‐IR (*n* = 34)	−0.42	[−1.24, 0.40]	NA	NA	−0.18	[−1.02, 0.65]
Systolic blood pressure (mm) (*n* = 34)	−1.69	[−6.08, 2.71]	NA	NA	−4.96	[−9.41, −0.50]
Diastolic blood pressure (Hg) (*n* = 34)	−2.28	[−6.747, 2.20]	NA	NA	−5.40	[−9.91, −0.88]
Total cholesterol (mmol/L) (*n* = 34)	−0.33	[−0.73, 0.08,]	NA	NA	−0.14	[−0.55, 0.27]
High‐density lipoprotein cholesterol (mmol/L) (*n* = 34)	−0.02	[−0.26, 0.21]	NA	NA	0.01	[−0.226, 0.25]
Low‐density lipoprotein cholesterol (mmol/L) (*n* = 34)	−0.37	[−0.78, 0.04]	NA	NA	−0.17	[−0.59, 0.24]
Lifestyle questionnaires						
Self Efficacy for Exercise (*n* = 42)	NA	NA	NA	NA	7.29	[−2.81, −17.40]
Maternal Antenatal/Postnatal Attachment Scale, MAAS/MPAS (*n* = 43)	NA	NA	NA	NA	0.02	[−0.15, 0.19]
Patient Health Questionnaire‐9 (*n* = 42)	NA	NA	NA	NA	0.50	[−1.981, 2.98]
24‐h dietary recall (Total Energy Intake), g/day (*n* = 32)	NA	NA	NA	NA	−268.99	[−520.44, −17.53]
International Physical Activity Questionnaire‐Short Form score (*n* = 44)	NA	NA	NA	NA	−316.20	[−2071.44,1439.04]
Average step count (*n* = 43)	NA	NA	NA	NA	854.91	[−288.30,1998.12]

*Note*: Linear mixed‐effects models were used, with a random intercept at the participant level to account for repeated measures. The repeatedly measured outcomes were the dependent variables, with the independent variables being trial arm, time point, and a trial arm by time interaction term to estimate the mean differences at each time point. Time 0 was the baseline time point and also the reference group. NA, not applicable, as data was not collected for that measure at that time point.

## 4 DISCUSSION

We conducted a feasibility study of an RCT to test the effectiveness of a digital DPI in Malaysian women with GDM. We found that it was feasible, acceptable, and safe to conduct the RCT. We achieved an acceptable rate of recruitment from the study population, in keeping with other DPI RCTs^30^ and over 90% retention and completion of follow up data.

The strengths of the study include the following: the study sample was representative of the distribution of the most ethnicities and socioeconomic diversity (in terms of income and education) in Malaysia; we recruited from a range of population density areas; we were able to adapt to distance working during Covid‐19 when recruitment was taking place. The study was set in a middle‐income nation where clinical trials, especially of complex interventions, are rarely conducted, which demonstrates acceptability by the research community and women with GDM.

The limitations of the study included first, the limited geographical area potentially reducing the representative of all Malaysian women, for instance the study was not designed to recruit from remote areas. Second, the methods used to collect uptake of the DPI was self‐report, therefore there may have been over‐estimation of the ‘dose’ reached but we were not able to report on app usage. Third, secondary to delays with Covid 19 lockdown, the follow up was reduced to 6 months rather than 12 months, therefore we were not able to estimate the 12 follow‐up rate. This limited the study's ability to assess the sustainability of behaviour change, longer‐term engagement with the intervention, and the persistence of effects beyond the early post‐partum period. There are some possible apparent differences between the arms on the distributions of ethnicity and education level, with more Malay participants with a tertiary education in the intervention arm, however, this is a small study and so some imbalances might be expected.

The study was not designed or powered to estimate an effect of the intervention, and therefore it was to be expected that there was no change in secondary outcomes from baseline to follow up. A contextual explanation was that the study was conducted during the Covid‐19 lockdown and most of the women participating may have been more focused on ensuring that they did not get infected or harm to their baby, so it is possible that they may not have accessed the DPI app as much as they would have done in a non‐pandemic situation. We also note that while the national lockdown ended in 2022, the clinical research environment remained constrained by infection control protocols and reduced patient volume until the complete lifting of healthcare SOPs in July 2023.

DPI for women post GDM are effective but less than for people at high risk of T2D in the general population. The reasons for this are complex and multi‐factorial. Studies of women with GDM in the UK suggest that education and information based DPIs may not be sufficient as the psychological sequelae of the diagnosis of GDM during pregnancy and postpartum are neglected.^31^, ^32^ These include depressive symptoms,^33^ medicalisation of pregnancy, societal stigma of blame and post‐partum disordered eating^32^, ^34^ which may be barriers to uptake of DPI. Our study cohort had on average mild depressive symptoms at baseline and at six month follow up and future observational studies are needed to estimate the extent to which these mediate dietary and physical activity behaviours. Our DPI had a mental health module which was requested during co‐production of the app by women with GDM; therefore, future DPIs may need to include techniques and content to address these psychological factors.

The successful delivery of this feasibility trial was underpinned by strong international collaboration. Knowledge transfer processes including trial planning, database setup, randomisation procedures, and the development of recruitment and consent workflows aligned with international standards were conducted primarily via virtual platforms. Delays arising from multiple waves of Covid‐19 and extended periods of partial movement control orders disrupted efficient follow‐up arrangements. While adaptive strategies such as online and telephone‐based participant interactions supported the delivery of the MI components of the intervention and demonstrated the feasibility of a digital behavioural support model, they might have limited interpersonal depth and therapeutic alliance crucial to behaviour change. Additionally, the reliance on self‐administered online outcome measures might have affected participation although we achieved good retention rates.

## 5 CONCLUSION

This feasibility study demonstrated that a RCT of a digital DPI for Malaysian women with GDM can be conducted safely and acceptably, with adequate engagement, recruitment and follow‐up. The trial also provided valuable organisational insights and lessons including successful co‐production of the intervention with patients in a middle‐income nation setting.

## AUTHOR CONTRIBUTIONS

Conceptualization (KI, BHC, AG, KG, SM, HRM) Data curation (SM, II, NHMS, BHC). Formal analysis (SM, II, KG) Funding acquisition (KI, BHC, AF, SM, HRM). Investigation (MB, KG, SM, II, AG, IPN, KI, BHC, SM, HRM). Methodology (KI, BHC, SM, HRM, NHMS, BNMY, NIB, IZI). Project administration (MB, KI, BHC). Supervision (KI, BHC, AF, KG). Validation (KI, BHC, KG) Writing—original draft (KI, MB, II, KG) Writing—review & editing (KI, MB, AF, II, KG, HRM, BHC).

## FUNDING INFORMATION

This work was supported by the Medical Research Council (MR/T018240/1) and Malaysia Partnerships and Alliances in Research (MYPAIR) Grant UK‐Malaysia: Joint Partnership Call on Non‐Communicable Diseases (Malaysia: JPT.S(BPKl)2000/011/06/05).^27^

## CONFLICT OF INTEREST STATEMENT

None to declare.

## Supporting information


Data S1.

## APPENDIX 1

Anthropometry, biochemical, psychological and lifestyle profiles of the participants of the MYGODDESS trial at T0 (baseline, 30 weeks gestation), T1 (36 weeks gestation), T2 (3‐month postpartum) and T3 (6‐month post‐partum).
