# Supplementary material for: A mobile phone application for the prevention of type 2 diabetes in Malaysian women with gestational diabetes mellitus (MYGODDESS): A feasibility randomised controlled trial
Source: Diabet Med. 2026 Mar 10;43(5):e70257. doi: 10.1111/dme.70257 (PMC13074133; doi:10.1111/dme.70257)
Supplement: Supplementary file 1 — Data S1. [file DME-43-e70257-s001.docx]

**Supplementary material 1**

Clinical variables consist of maternal and neonatal variables collected at 3 months postpartum (time 2). These outcomes were tabulate descriptively which may also be potential predictors or modifiers of postpartum outcomes at the later time points, which could be helpful to consider for a larger trial.

| **Variable** | **Control group, n (%)/ mean (SD)** | **Intervention group, n (%)/ mean (SD)** | **Overall, n (%)/ mean (SD)** |
| --- | --- | --- | --- |
| **Hypertension** |  |  |  |
| Yes | 0 (0.0) | 0 (0.0) | 0 (0.0) |
| No | 27 (52.9) | 24 (47.1) | 51 (100.0) |
| **Hypercholesterolemia** |  |  |  |
| Yes | 0 (0.0) | 0 (0.0) | 0 (0.0) |
| No | 27 (52.9) | 24 (47.1) | 51 (100.0) |
| **Place of antenatal care** | | | |
| Klinik Kesihatan Seri   Kembangan | 7 (13.7) | 11 (21.6) | 18 (35.3) |
| Klinik Kesihatan Puchong   Batu 14 | 13 (25.5) | 11 (21.6) | 24 (47.1) |
| Klinik Kesihatan   Putrajaya Presint 9 | 7 (13.7) | 2 (3.9) | 9 (17.6) |
| **Place of delivery** | | | |
| In Selangor | 23 (45.1) | 20 (39.2) | 43 (84.3) |
| Outside Selangor | 4 (7.8) | 4 (7.8) | 8 (15.7) |
| **Delivery method** | | | |
| Spontaneous vaginal | 12 (23.5) | 14 (27.5) | 26 (51.0) |
| Vacuum | 3 (5.9) | 5 (9.8) | 8 (15.7) |
| Caesarean | 4 (7.8) | 1 (2.0) | 5 (9.8) |
| Emergency caesarean | 8 (15.7) | 4 (7.8) | 12 (23.5) |
| **Pregnancy complications** | | | |
| No complication | 24 (47.1) | 24 (47.1) | 48 (94.1) |
| Pre-labour rupture   membrane | 1 (2.0) | 0 (0.0) | 1 (2.0) |
| Others* | 1 (2.0) | 0 (0.0) | 1 (2.0) |
| More than 2   complications | 1 (2.0) | 0 (0.0) | 1 (2.0) |
| **Birth complications** | | | |
| No complication | 24 (47.1) | 22 (43.1) | 46 (90.2) |
| Premature baby | 2 (3.9) | 0 (0.0) | 2 (3.9) |
| Others | 1 (2.0) | 2 (3.9) | 3 (5.9) |
| **Neonatal data** |  |  |  |
| **Congenital anomaly** | | | |
| Yes | 0 (0.0) | 0 (0.0) | 0 (0.0) |
| No | 27.00 | 24 (47.1) | 51 (100.0) |
| **Congenital pneumonia/heart defects** | | | |
| Yes | 0 (0.0) | 1 (2.0) | 1 (2.0) |
| No | 27 (52.9) | 23 (45.1) | 50 (98.0) |
| **Admission to neonatal intensive care, special care or qualified on ward** | | | |
| Yes | 15 (29.4) | 11 (21.6) | 26 (51.0) |
| No | 12 (23.5) | 13 (25.5) | 25 (49.0) |
| **Birth weight (g)** | 2928.5 (413.7) | 3100.4 (336.2) | 51 (100.0) |
| **APGAR score 5 minutes (0-3/4-6/7-10)** | 9.9 (0.3) | 10.0 (0.0) | 51 (100.0) |
| **Length of stay (days)** | 2.0 (3.0) | 2.0 (2.0) | 51 (100.0) |
| *anaesthethic complication, antepartum haemorrhage, cord prolapse, deep vein thrombosis, disseminated intravascular coagulation, embolism, post-partum haemorrhage | | | |
